# P03-05 State of the Evidence of Active Living among Children and Youth in India: A Scoping Review Informing the Global Matrix 4.0

**DOI:** 10.1093/eurpub/ckac095.041

**Published:** 2022-08-29

**Authors:** Jasmin Bhawra, Tarun R Katapally

**Affiliations:** 1 University of Saskatchewan, London, Canada; 2 Western University, London, Canada

**Keywords:** physical activity, active living, children, youth, international, India, global, Global Matrix 4.0

## Abstract

**Background:**

There is strong evidence of physical inactivity's link to global disease and economic burden. Physical inactivity among Indian children and youth has particular consequences for the global economy, as youth in India make up a substantial proportion of the world's workforce. As part of the 60-country Active Healthy Kids Alliance Global Matrix 4.0 initiative, a systematic scoping review was conducted to appraise the current state of evidence of active living among children and youth in India.

**Methods:**

A systematic search of peer-reviewed and grey literature published over the last decade was conducted for 11 indicators of active living: overall physical activity, organized sport participation, active play, active transportation, sedentary behavior, family and peers, school programs and policies, community and built environment, government strategies, physical fitness, and yoga. Data sources included national, state (i.e., province) and city-level surveys, as well as primary data from ongoing longitudinal studies. Relevant grey literature, including government reports and school board policies, were also reviewed.

**Results:**

Physical activity levels vary widely across India, with children and youth in rural settings accumulating greater moderate-to-vigorous activity and lower screen time compared to their urban counterparts. The majority of Indian children and youth report active transportation, however; they are not meeting recommended physical activity and sedentary behaviour guidelines. Despite the availability of many non-profit and organized programs, several indicators of active living including organized sports, active play, and yoga programming have not been evaluated for uptake or impact. Physical activity type and levels varied significantly across gender and socioeconomic status, with girls belonging to lower socioeconomic status having the greatest disadvantage due to cultural and safety perceptions.

**Conclusions:**

While the vast majority of Indian children and youth are not accumulating recommended physical activity levels, there are encouraging signs of active transportation and active play?a phenomenon that needs to be further explored in India and other high-, middle- and low-income countries. The findings point to widespread disparities in access to active living resources and infrastructure between urban and rural settings. Targeted programs, policies, and resource allocation are necessary to improve built environment and safety for children and youth.

